# Analysis of pain level in cases treated with Invisalign aligner: comparison with fixed edgewise appliance therapy

**DOI:** 10.1186/s40510-014-0064-7

**Published:** 2014-11-22

**Authors:** Koji Fujiyama, Tadashi Honjo, Makoto Suzuki, Shinya Matsuoka, Toru Deguchi

**Affiliations:** Fujiyama Orthodontic Clinic, Kitanokami hakubai-cho 66, Kita-ku, Kyoto, 603-8325 Japan; Department of Oral Maxillofacial Surgery, University of Tottori School of Medicine, Nishi-machi 36-1, Yonago, Tottori, 683-8504 Japan; Matsuoka & Yamazaki Orthodontic Clinic, Chuou-ku 6-7-16, Tokyo, 104-0061 Japan; Division of Orthodontics, The Ohio State University College of Dentistry, 305 W. 12th Avenue, Columbus, OH 43210-1267 USA

**Keywords:** Invisalign, Pain, Tooth movement, Edgewise appliance

## Abstract

**Background:**

The aim of this study was to evaluate and compare the difference in the level of pain using the visual analog scale (VAS) between cases treated with the edgewise appliance and Invisalign. In addition, the cause of pain and discomfort in the Invisalign cases was identified.

**Methods:**

The sample consisted of 145 cases for the edgewise group (EG; *n* = 55), Invisalign group (IG; *n* = 38), and edgewise and Invisalign group (EIG; *n* = 52). VAS scores were collected during the first three stages (first stage: 0 to 7 days, second stage: 14 to 21 days, and third stage: 28 to 35 days) and at the end of the treatment (overall VAS score). Evaluation of the cause of pain was categorized into three different types of problem (category 1: non-smoothed marginal ridge or missing materials, category 2: deformation of attachments, and Category 3: deformation of the tray). Statistical comparison of VAS scores between groups was performed by two-way analysis of variance.

**Results:**

A significantly higher VAS score was observed at 3 and 4 days after, at 1, 2, and 3 days after, and at 2 and 3 days after in stages 1, 2, and 3, respectively, in EG compared to EIG and IG. A significant difference was observed in overall VAS scores between EG and IG in intensity of pain, number of days that pain lasted, and discomfort level. Only intensity of pain resulted in a significant difference between EG and EIG. Most of the causes of problem in the Invisalign cases were deformation of the tray.

**Conclusions:**

Invisalign may offer less pain compared to the edgewise appliance during the initial stages of treatment. In the use of Invisalign, deformation of tray must be carefully checked to avoid pain and discomfort for the patients.

## Background

Pain and discomfort during orthodontic tooth movement are the most negative concerns for treatment [1]. In general, pain adversely affects patients' quality of life (QOL) [2,3]. The pain experienced during orthodontic treatment occurs several hours after the orthodontic force is applied, and the most pain is observed after 24 h and decreases to near-baseline level after 7 days [4,5]. The first 7 days would be the most painful and critical for patients through the entire treatment period. Therefore, it would be a great benefit for patients if we could reduce the pain during orthodontic treatment.

There have been several studies that have analyzed the level of pain in different types of orthodontic appliances. In most of the past studies, there was no significant difference in the level of pain treated between fixed appliances such as with self-ligation, lingual, or conventional brackets [6,7]. On the other hand, fixed appliances resulted in a significantly higher pain level than removable appliances [8]. Higher values of the intensities of pressure, tension, pain, and sensitivity of the teeth were reported in patients treated with fixed appliances in contrast to those wearing functional appliances. A higher pain level may be observed with fixed appliances than with the removable type of appliances in general.

In recent years, aligners [9] have become one of the most growing orthodontic treatment modalities, especially Invisalign [10,11]. Many adult patients seek for esthetic appliances such as clear brackets, lingual appliances, and Invisalign. There are several advantages of using Invisalign over other appliances such as superior esthetics, comfort, and oral hygiene. In addition to these advantages, pain is also suggested to be less compared to conventional brackets [12]. However, there are two past studies that analyzed the pain value in Invisalign patients that had contrary results compared with conventional brackets [12,13]. On the other hand, difficulty in finishing [14] and limitation in treating extraction cases [15] have been known as the disadvantages of Invisalign treatment.

Therefore, we evaluated the level of pain using the visual analogue scale (VAS) between groups that were treated with the conventional edgewise appliance (EG), Invisalign (IG), and both the edgewise appliance and Invisalign (EIG). Furthermore, we identified the cause of pain and/or discomfort in the Invisalign groups. Our hypothesis was that there would be a significant difference in the level of pain between these groups.

## Methods

EG consisted of 55 adult patients (35 females and 20 males, mean age 26.45 ± 5.45 years), IG consisted of 38 adult patients (28 females and 10 males, mean age 26.64 ± 5.69 years), and EIG consisted of 52 adult patients (33 females and 19 males, mean age 25.24 ± 6.51 years). Consecutive patients were prospectively collected from one private orthodontic clinic. Exclusion criteria were as follows: under 18 years of age, complex or surgery cases, and cases that were only treated in one jaw. This study was approved by the Ethics in Research Committee of the University of Tottori. All patients received informed consent and understood and agreed to the purpose of this study.

In EG, all cases were treated with a straight wire appliance with .018 slot edgewise brackets. In IG, only Invisalign aligners were used for the treatment. Patients were instructed to wear the aligner for a minimum of 20 h per day. In EIG, all cases had experienced both the straight wire edgewise appliance and Invisalign aligners. The percentage of extraction cases was 23.6% (13/55), 18.4% (7/38), and 18.5% (10/52) for EG, IG, and EIG, respectively.

Subjects were instructed to mark their level of pain on a 10-cm VAS during the three stages of treatment. The mark was measured in millimeters with a 10-cm ruler from the left side. Each millimeter was given a VAS score of 1 such that a score of 0 at the left end of the scale indicated no pain, a score of 100 at the right end of the scale indicated maximum pain, and a score of 50 in the center of the scale indicated moderate pain. This was explained to each patient before the study.

The first stage of collecting the VAS score was at 60 s, at 6 and 12 h, and at 1, 2, 3, 4, 5, 6, and 7 days after the appliance delivery (changing wires in EG and new trays in IG). The second stage was at 3 weeks after the appliance delivery, and the patients were also asked to mark from 60 s to 7 days after. The third stage was at 5 weeks after the appliance delivery. We changed the wire in a 2-week interval instead of 4 weeks (which is suggested to be the usual treatment interval in edgewise cases) in order to match the treatment interval with that of IG. For EIG, the average VAS score was measured after the delivery of both appliances (edgewise appliance and Invisalign trays) during the first three stages of each treatment. All cases in EIG initially had edgewise treatment followed by Invisalign treatment. The overall VAS score was also taken immediately after the treatment was finished in all three groups. Since patients in EIG have experienced both appliances, we also collected the overall VAS score for both the edgewise appliance and Invisalign at the end of the treatment.

**Figure 1:**
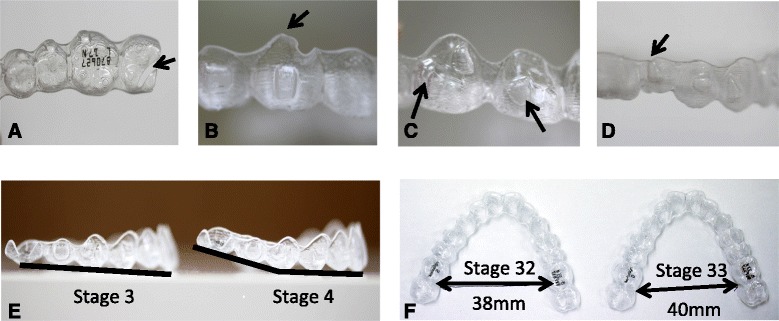
**Three categories of reasons for pain or discomfort.** Category 1 indicates trays with some materials missing (arrow in **(A)**) or non-smooth edges (arrow in **(B)**). Category 2 indicates deformation of the attachments at the occlusal margin (arrows in **(C)**) and at the gingival margin (arrow in **(D)**). Category 3 indicates deformation of the tray in vertical dimension **(E)** and in transverse dimension **(F)**.

In addition, we have analyzed the reasons for the pain or discomfort level beyond VAS score 50 (>moderate) and identified the common problems in IG. We divided the reasons for pain or discomfort into three categories (category 1: missing materials in the occlusal surface or non-smoothed marginal ridge, category 2: deformation of attachments, and category 3: deformation of the tray) (Figure 1).

Two-way analysis of variance (ANOVA) for repeated measures and Bonferroni *post hoc* tests were used to compare between groups by Statistical Package for the Social Sciences (SPSS) computer software for Windows. Non-significant values were defined as *p* < 0.05.

## Results

### VAS scores during the initial three stages

**Figure 2:**
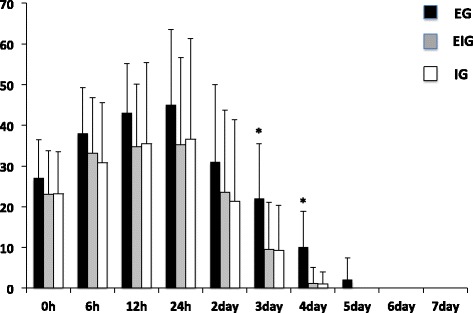
**Graph showing changes of VAS score during the first stage (h: hours).** *Significant difference compared with EIG and IG; *p* < 0.05.

At stage 1, the peak VAS score was observed at 24 h in all three groups and lasted for 4 days in EIG and IG and for 5 days in EG (Figure 2). EG was significantly higher than EIG and IG at 3 and 4 days after.

**Figure 3:**
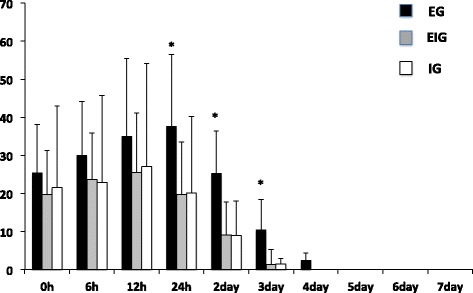
**Graph showing changes of VAS score during the second stage (h: hours).** *Significant difference compared with EIG and IG; *p* < 0.05.

**Figure 4:**
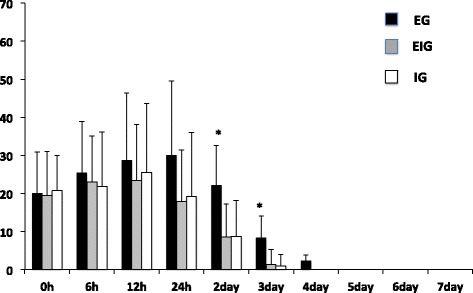
**Graph showing changes of VAS score during the third stage (h: hours).** *Significant difference compared with EIG and IG; *p* < 0.05.

At stages 2 (Figure 3) and 3 (Figure 4), the peak VAS score was seen after 12 h in EIG and IG and lasted for 3 days in EIG and IG and for 4 days in EG. A significantly higher VAS score was observed in EG compared to EIG and IG at 2 and 3 days after.

### Overall VAS score

**Figure 5:**
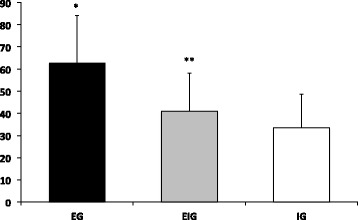
**Overall VAS score for intensity of pain.** *Significant difference compared to EIG and IG; *p* < 0.05. **Significant difference compared to IG; *p* < 0.05.

In the overall VAS score for intensity of pain, a significantly higher VAS score was observed in EG compared with both EIG and IG (Figure 5).

**Figure 6:**
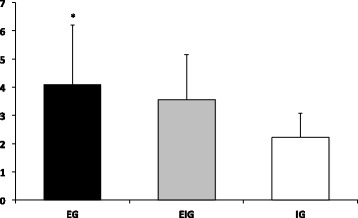
**Overall number of days that pain lasted.** *Significant difference compared to IG; *p* < 0.05.

The number of days that pain lasted was significantly longer in EG compared to IG (Figure 6).

**Figure 7:**
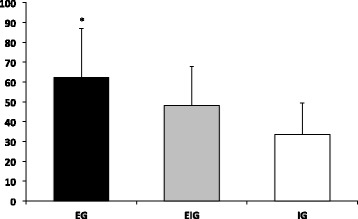
**Overall discomfort level.** *Significant difference compared to IG; *p* < 0.05.

The overall VAS score for the discomfort level resulted in a significantly higher score for EG compared to IG (Figure 7).

**Figure 8:**
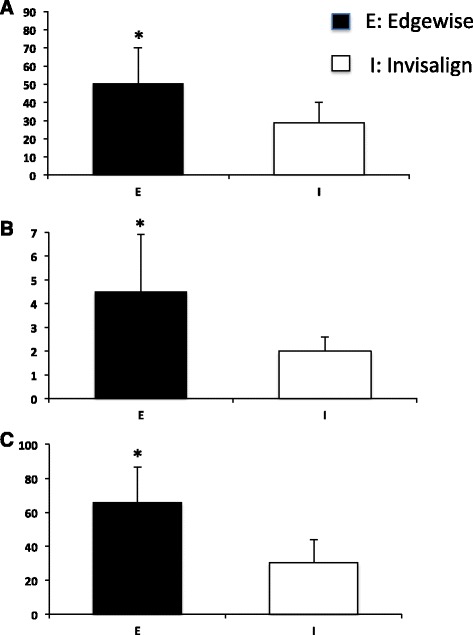
**Overall VAS scores in IEG.** Overall VAS scores for intensity of pain **(A)**, number of days that pain lasted **(B)**, and discomfort level **(C)** in IEG. *Significant difference between two groups; *p* < 0.05.

For EIG, significantly higher scores were observed in the edgewise appliance compared to Invisalign in all three variables (intensity of pain, number of days, and discomfort level) (Figure 8).

### Reasons for pain and/or discomfort in IG

Approximately 11% (10/90 cases) of Invisalign cases complained significant pain or discomfort, and approximately 3% (369/12,311) of the total trays had problems. Reasons for pain or discomfort resulted in 23, 25, and 316 cases in categories 1, 2, and 3, respectively (Table 1).

**Table 1:** **Percentage of deformed aligners**

	**Category 1**	**Category 2**	**Category 3**	**Total**
Poor conformity aligners	0.2% (23/12,311)	0.2% (25/12,311)	2.6% (316/12,311)	3.0% (369/12,311)

## Discussion

In this study, we analyzed the VAS scores not only in the first week after the appliance delivery but also after 3 and 5 weeks of appliance adjustments. At the first stage, the peak VAS score was observed 1 day after and gradually decreased in all three groups. Generally, the pain increases few hours after placement of the initial arch wire, peaks at 24 h, and decreases to almost baseline levels at 7 days [4,5]. Ngan et al. report that on surveying pain levels 4 h, 24 h, and 7 days after the insertion of arch wires and separators, pain was greatest when using both devices after 24 h [5]. Harazaki and Isshiki implemented a questionnaire survey regarding the time during which pain was felt by patients having the initial wire inserted to an attachment device fitted to all their teeth and reported that pain occurred after between 3.4 and 3.5 h, peaked at around 24 h, and disappeared a week later [16]. In each of these cases, the pain threshold was at its lowest around 24 h after the application of orthodontic force, after which gradual recovery was noted. In this study, the pain disappeared 5 days after in EIG and IG but not until 6 days after in EG. All VAS scores decreased while the stage progressed.

In stage 1, more pain was observed 3 and 4 days after, but there was also more pain after 1 day in stage 2 and after 2 and 3 days in stage 3 in EG compared to EIG and IG. This indicates that the intensity of pain may not be different before 24 h of appliance adjustment, but after 2 to 3 days, the edgewise appliance may produce more pain than Invisalign. Also, the edgewise appliance may cause prolonged pain compared to Invisalign. This is consistent with a similar past study which indicated that fixed appliance subjects reported more pain than Invisalign subjects [12]. Others also indicated that fixed appliances caused more pain or discomfort to patients than removable appliances [8,17].

One of the unique data of this study is that we were able to compare the pain level between the edgewise appliance and Invisalign in the same patient (EIG). During the initial stages of treatment, EIG resulted in a similar VAS score to that of IG. The combination approach by initial alignment with the edgewise appliance and later with Invisalign tends to have less pain than just with edgewise treatment. The overall VAS scores for EIG resulted in significantly less pain, duration, and discomfort during the Invisalign treatment period than during the initial edgewise treatment. Therefore, patients who experienced both appliances prefer Invisalign than edgewise treatment from a pain and discomfort point of view. However, the limitation of this result is that since all EIG patients experienced the edgewise appliance prior to Invisalign, the initial edgewise treatment may have masked the intensity of pain by the use of Invisalign. Thus, comparison of VAS between the group that has the edgewise appliance initially and the group that started with Invisalign must be performed to clarify this problem.

Our overall results collected after the treatment also showed significantly more and long-lasting pain in EG than in IG. However, there was no significant difference in pain duration and discomfort between EG and EIG. Thus, patients may feel less pain with Invisalign, but the duration of pain and discomfort level seems to be the same. Since several patients complained of pain or discomfort, we further identified the problems with Invisalign trays. As a result, most of the reasons for pain or discomfort in the Invisalign cases were deformation of trays. Some patients experienced pain other than the four categories such as pain related to the change in the gingival morphology due to the eruption of third molars, swelling of the gingiva due to inefficient oral hygiene, and inefficient use of aligner (bad cooperation) which were not included in the present analysis. Recently, since there was a change in the material of the tray from EX30 to LE30, which is softer and has more flexibility, this problem should be greatly improved.

One of the limitations of this study was the different extraction ratios among groups. The extraction ratio was quite similar between IG and EIG; however, EG has a higher extraction rate (approximately 5% higher) compared to the other two groups. Since extraction requires a surgical procedure, it might have resulted in higher VAS scores compared to non-extraction therapy. However, we suggest that a 5% difference would not have a significant impact on the results of our study. Future studies such as randomized clinical trials with more controlled selection of the sample are required to elucidate this problem.

Brown and Moerenhout indicated that adolescents, pre-adolescents, and adults varied in their pain reports during orthodontic treatment, with adolescents reporting the most pain. In this study, the average age was very similar among the three groups; thus, there should not be any influence on our results on age among groups. On the other hand, the male:female ratio was 20:35, 10:28, and 19:33 for EG, IG, and EIG, respectively. IG had a higher female ratio (73.7%) compared to EG (63.6%) and EIG (63.5%). However, since a lower VAS score was observed in IG (with a higher female ratio), we indicate that there was no significant impact on the results of our study.

## Conclusions

In conclusion, Invisalign results in less pain compared to the edgewise appliance approximately 3 days after delivery or adjustment during the initial stage (approximately 5 weeks) of the treatment. Overall, at the end of the treatment, patients also observed that less pain was felt with Invisalign treatment compared to the edgewise appliance. In addition, the major cause of pain or discomfort in the Invisalign cases is tray deformation. Thus, Invisalign offers less pain compared to the traditional edgewise treatment; however, problems such as tray deformation must be carefully checked in the use of Invisalign.
